# AntigenApp: a laboratory data management system for nanobody generation and sequence analysis

**DOI:** 10.1093/bioinformatics/btaf642

**Published:** 2025-12-01

**Authors:** Alexander L R Lubbock, Lauren E -A Eyssen, Kelly Parker, Mark Basham, Laura A Shemilt, Raymond J Owens

**Affiliations:** The Rosalind Franklin Institute, Harwell Science Campus, Didcot, OX11 0QX, United Kingdom; The Rosalind Franklin Institute, Harwell Science Campus, Didcot, OX11 0QX, United Kingdom; The Rosalind Franklin Institute, Harwell Science Campus, Didcot, OX11 0QX, United Kingdom; The Rosalind Franklin Institute, Harwell Science Campus, Didcot, OX11 0QX, United Kingdom; The Rosalind Franklin Institute, Harwell Science Campus, Didcot, OX11 0QX, United Kingdom; The Rosalind Franklin Institute, Harwell Science Campus, Didcot, OX11 0QX, United Kingdom; Division of Structural Biology, Nuffield Department of Medicine, University of Oxford, OX3 7BN, United Kingdom

## Abstract

**Motivation:**

Nanobodies are single domain antibodies derived from the unique heavy chain-only immunoglobulins of the camelid family, and have broad applications in biosciences, including as imaging and diagnostic agents. The generation of nanobodies is a multi-stage experimental process for which we have developed AntigenApp a laboratory data management system for nanobody generation and sequence analysis.

**Results:**

AntigenApp is a web application and database built to capture data generated during all steps of the nanobody discovery pipeline and to provide built-in analysis capabilities of the experimental results. This includes enzyme-linked immunosorbent assay optical density measurements, sequencing results, nanobody domain labelling, and search/clustering capabilities using protein BLAST. The tool incorporates a standardized naming convention that is automatically applied to nanobodies stored in the database and we envisage the tool being of interest to the wider nanobody community for in-house use.

**Availability and implementation:**

AntigenApp is available from https://github.com/rosalindfranklininstitute/antigen-app under the Apache 2.0 licence and published https://doi.org/10.5281/zenodo.17397055.

## 1 Introduction

Members of the camelid family produce a subset of antibodies that consist of heavy chains that lack CH1 domains and hence associated light chains. The so-called variable heavy domains (VHH) of these immunoglobulins contain the binding paratope and can be produced as single domain antibodies or nanobodies (Muyldermans 2013). The small size and relatively low chemical complexity of nanobodies compared to typical antibodies, has led to applications in bioimaging (Rashidian and Ploegh 2020), diagnostics, and as therapeutics (Jin *et al.* 2023). Antigen-specific nanobodies are typically selected from VHH expression libraries using various *display technologies* in multi-step experimental pipelines. Recording experimental (meta)data, through the different steps of the process, including nanobody selection, is crucial for building a scalable workflow and a knowledgebase for future datamining. Therefore, we designed a laboratory data management system, AntigenApp, to capture, store and share information about experiments replacing shared spreadsheets, that were difficult to scale as the rate of data generation increased. Several *in silico* operations in the pipeline were also manual and time-consuming, including the collation of selected hits from several plates into a single 96-well plate layout for sequencing, converting the sequencing results into a format suitable for bioinformatics analysis, and analysing these sequencing result sets individually (and storing the resulting files). Functionality built into AntigenApp stores data within a structured relational database and ensuring data quality standards are upheld. Based on enzyme-linked immunosorbent assay (ELISA) data and user-defined thresholding of the colorimetric or fluorescent readout from the ELISA, 96-well plate layouts for sequencing are generated automatically, and bioinformatics analysis is now an automated step when sequencing results are uploaded. Therefore, the primary benefits of using AntigenApp are in data standardization and validation during entry, auditability, team-wide visibility and collaboration, and automation/time-savings from reducing manual steps and increased integration. Nanobody discovery often yields proprietary or patentable data, so we designed AntigenApp to run as separate in-house instances rather than as a single centralized service. We envisage it being useful to other laboratories and facilities and have released the software under an open-source Apache 2.0 license for all to use. In the following sections, we discuss the technical implementation details of the software, detail its core features and present our conclusions.

## 2 Implementation and functionality

**Figure 1.**
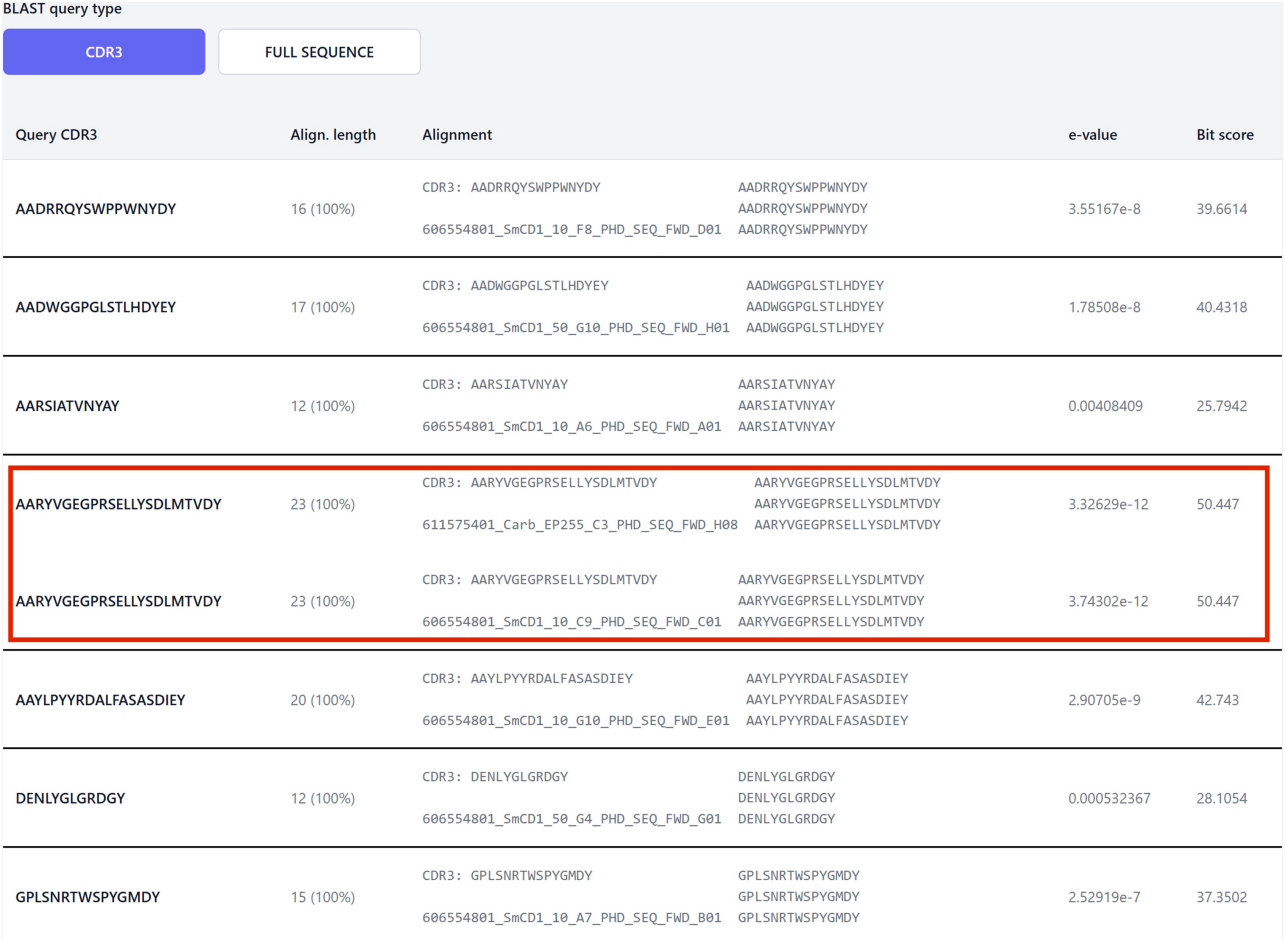
Results of alignment-based search that was generated from the exemplar data against previous experiments. The CDR3s from only seven of the eight hits appear in this search as one of the clones was unproductive and was automatically omitted. Nanobodies with the same CDR3 sequence are boxed in red.

AntigenApp is split into a backend, consisting of a PostgreSQL relational database and application programming interface (API), and a frontend, which runs in a web browser and serves as the user interface (Fig. 1, available as supplementary data at *Bioinformatics* online). The backend is written in Python using Django (djangoproject.com) and the Django REST Framework (django-rest-framework.org). The frontend is written in JavaScript using React (react.dev). The software is deployed in containers and runs on Kubernetes (kubernetes.io). Further implementation details are included in Material 2, available as supplementary data at *Bioinformatics* online and Fig. 1, available as supplementary data at *Bioinformatics* online.

The AntigenApp interface has sections relating to major data classes, including Projects, Antigens, Llamas, Sequencing and Nanobodies (Fig. 2, available as supplementary data at *Bioinformatics* online). These entities map to the data types generated by the experimental pipeline, described previously (Eyssen *et al.* 2024). We create entities in AntigenApp for our data in the following order: *antigens, llama, cohort, project, library, ELISAs* and *sequencing runs*. For the purposes of this software, each *llama* can be immunized with one or more *antigens* as part of a *cohort*. Each *antigen* can either be entered manually or via a UniProt ID (The UniProt Consortium 2015); for the latter, the name, sequence, and molecular mass are automatically populated. A *project* contains a set of *libraries*, where each *library* maps one-to-one with a *cohort*; in cases where a project has re-used a cohort more than once, e.g. to look at cross-reactive binding (Sørensen *et al.* 2023), this is termed a *sub-library*. Each library (or sub-library) contains one or more *enzyme linked immunosorbent assays (ELISAs)*, which are in vitro experiments to determine nanobody binding to a single target *antigen*, measured by optical density (arbitrary units). A *sequencing run* is then created, which takes forward promising *nanobody* candidates using a graphical thresholding system. Note that *nanobodies* from multiple *ELISAs* can be combined within a sequencing run, across one or more 96-well plates, to optimize efficiency and reduce cost. Sequencing results are then uploaded to AntigenApp in FASTA format, where they are processed through *IMGT V-QUEST* (Brochet *et al.* 2008), an online bioinformatics platform which converts each nucleotide sequence to an annotated amino acid sequence. These results are viewable as a table within AntigenApp, where such attributes as presence of a start codon and sequences for the *complementarity-determining regions (CDRs)* are shown. Access is also provided to the IMGT results file in Adaptive Immune Receptor Repertoire (AIRR) format (Vander Heiden *et al.* 2018).

All nanobody sequences (both DNA and protein) are stored in the AntigenApp database and the sequencing run results view also incorporates the Basic Local Alignment Search Tool for Proteins (protein BLAST, BLASTp) tool. The data can then be searched by going to the sequencing results list, choosing a sequencing plate run and then selecting the BLAST tab. This tool performs an alignment-based search (by full sequence or CDR3) of the AntigenApp database of experimental sequences (i.e. a many-to-many search). The results are returned in an annotated, sorted table in which any CDR3 sequence matches to nanobodies already in the AntigenApp database are shown (Fig. 1) indicating potential cross-reactivity. The AntigenApp database can also be searched with either individual full or CDR sequences from external publicly available sources, by selecting the ‘BLAST by sequence’ button under the sequencing tab.

The frontend interface allows users to create, read, update and delete objects from the database. Data entry takes place via web forms and file upload. Data entry operations are logged in the database and are viewable as an audit log within the user interface, allowing users to see who edited what and when for full transparency.

We illustrate AntigenApp through an exemplar nanobody discovery project. The objective of the study was to identify nanobodies to the aspartate protease, Cathepsin D1, from *Schistosoma mansoni* (Uniprot: G4VEV6), a potential drug target for treating Schistomiasis (Morales *et al.* 2008). The exemplar dataset is provided in Material 3, available as supplementary data at *Bioinformatics* online and Fig. 3, available as supplementary data at *Bioinformatics* online.

Both the AntigenApp software and exemplar dataset can be tried locally via Docker Compose, which is available for Windows, Mac and Linux. Instructions are provided in the README file. Deploying in a multi-user ‘production’ environment has more considerations around authentication, security, storage, monitoring and backups; see Material 4, available as supplementary data at *Bioinformatics* online.

## 3 Conclusion

AntigenApp is a laboratory data management and analysis platform for nanobody generation and sequence analysis. In addition to storing experimental and sequencing results, logging updates and changes, and making data searchable by a team, it also provides sequence search and analysis capabilities. Additional benefits come from the time-saving automation from having IMGT-VQUEST running automatically when new data are uploaded and by having BLASTp integrated into the software for private, local use. We plan to incorporate further experimental results into AntigenApp including the expression yield and binding affinity of nanobodies using the same approach for integrating the phage ELISA and sequencing data. We anticipate that the open-source software will be of interest to teams generating or working with nanobodies and can be customized and extended to fit individual team needs.

## Supplementary Material

btaf642_Supplementary_Data
