# Supplementary material for: AntigenApp: a laboratory data management system for nanobody generation and sequence analysis
Source: Bioinformatics. 2025 Dec 1;41(12):btaf642. doi: 10.1093/bioinformatics/btaf642 (PMC12701793; doi:10.1093/bioinformatics/btaf642)
Supplement: btaf642_Supplementary_Data [file btaf642_supplementary_data.docx]

# AntigenApp: a laboratory data management system for nanobody generation and sequence analysis

# Supplementary Material

## S1 Glossary

**Antibody**: A Y-shaped protein produced by the immune system that recognises and binds to specific antigens, helping to neutralise pathogens such as bacteria and viruses.

**BLASTp (Basic Local Alignment Search Tool for Proteins)**: A computational algorithm that compares a protein sequence against a database of known protein sequences to identify similarities, aiding in functional annotation and comparative analysis.

**CDR/CDR3 (complementarity-determining region)**: CDRs are hypervariable regions in antibodies and T cell receptors responsible for antigen recognition. CDR3, the most variable of these regions, plays a crucial role in defining binding specificity and affinity.

**Display Technologies**: The display of antibodies, nanobodies and other antibody fragments on the surface of, most commonly, phage and yeast. Less common are bacteria, mammalian, ribosome and mRNA display.

**ELISA** (**Enzyme-Linked Immunosorbent Assay)**: A laboratory technique used to detect and quantify specific proteins and/or their binding affinity for a target, such as antibodies or antigens, using enzyme-linked antibodies and a colourimetric or fluorescent readout.

**IMGT V-QUEST**: A bioinformatics tool provided by the International ImMunoGeneTics (IMGT) database, used for analysing and annotating immunoglobulin (Ig) and T cell receptor (TCR) sequences, including gene identification and characterisation of complementarity-determining regions (CDRs).

**Nanobody**: A small, single-domain antibody derived from camelid antibodies, typically with high stability and strong antigen-binding affinity.

**Sequencing run**: A single execution of a DNA sequencing process on a sequencing platform, generating raw nucleotide sequence data for analysis.

## S2 Extended description of software implementation

The AntigenApp technology stack is shown in Figure S1. The navigational structure of the AntigenApp user interface is shown in Figure S2.

### Data schema and storage

The database schema is implemented as Python classes which are automatically mapped to the database using Django’s object-relational mapper (ORM). Uploaded files are stored in S3-compatible storage, in our case using an Amazon Web Services (AWS) S3 bucket.

### Continuous integration and continuous deployment (CI/CD)

The system runs in containers which we run on Docker (docker.com) for local development and Kubernetes (kubernetes.io) in production. New container images are built automatically using GitHub Actions and deployed to our container registry (quay.io/organization/rosalindfranklininstitute). We run ArgoCD (argo-cd.readthedocs.io) as a continuous deployment platform for Kubernetes to get automatic updates.

Figure S1: AntigenApp implementation and technology stack. AntigenApp consists of a Python backend and JavaScript frontend, utilising the Django REST Framework and React respectively. Data are stored in a relational database (PostgreSQL) and object storage (Amazon Web Services S3). Authentication is provided via Oauth2-proxy and Keycloak. Error monitoring is provided by Sentry. The software uses continuous integration and deployment to test, build and run the software, which uses GitHub Actions, quay.io, ArgoCD and Kubernetes.

Figure S2: AntigenApp navigational structure for managing and searching data. Screenshots show each of the major user interface elements for managing different data classes; arrows show how to navigate between them.

## S3 Description of demonstration dataset

Nanobody discovery was carried out for SmCD1 (Uniprot: G4VEV6, residues 15-385) as described in (Eyssen *et al.*, 2024) and the progress depicted in Figure S3. Briefly, a llama was immunised with recombinant SmCD1 and a library containing approximately 1x106 nanobody VHH repertoire was produced. At both panning rounds (50 and 10nM), 93 clones were picked and assayed using an anti-M13 ELISA which identified 8 potential hits. Following the small-scale expression protocol, only 6 of the 8 clones were successfully expressed and purified of which only 3 showed binding to the SmCD1 antigen. After large scale expression and purification of the 3 nanobodies, a titration ELISA demonstrated that only 2 of the 3 clones were positive SmCD1 binders. Only 1 of these nanobodies successfully formed a complex with SmCD1 and its structure was solved by X-ray crystallography (manuscript in preparation).


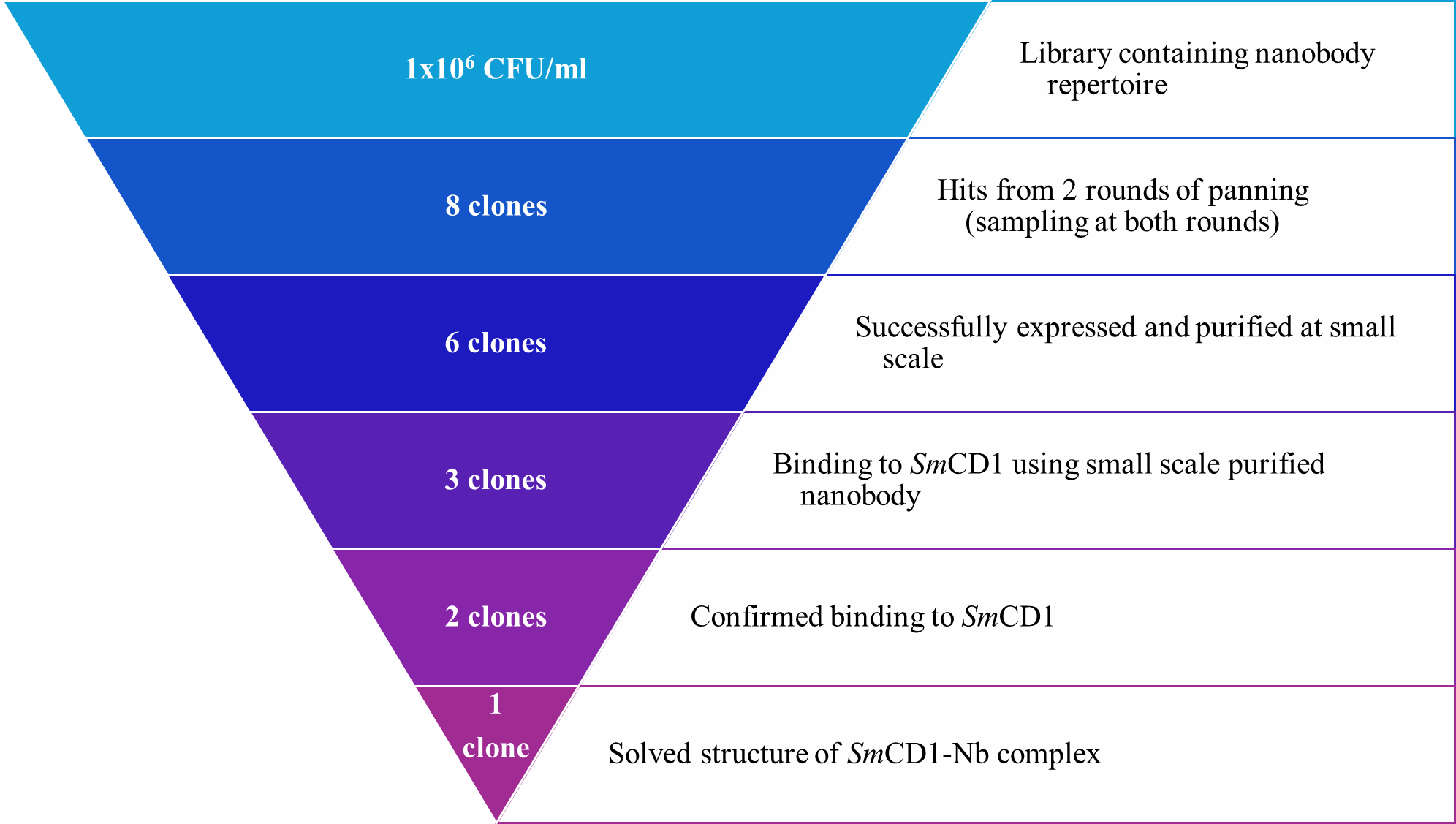


Figure S32: Process of the nanobody discovery pipeline for SmCD1 from immune library to nanobody application as a crystallisation chaperone.

## S4 Considerations for deploying the software in a multi-user environment

### Authentication

AntigenApp relies on HTTP headers to provide authentication, specifically a username in X-Auth-Request-Preferred-Username and an email address in X-Auth-Request-Email. Care should be taken to ensure these headers are set by a reverse proxy server so that they can’t be set/spoofed remotely. We use Oauth2-proxy to integrate with Keycloak using the OpenID Connect (OIDC) protocol. Oauth2-proxy also supports other authentication systems including social media logins, which are described in its documentation.

### Error monitoring

AntigenApp is integrated with Sentry, which can be self-hosted or used as a service. Entering a Sentry API key into the configuration will enable capture of any issues within the software itself. If you discover any issues with the software, please report these on our GitHub at <https://github.com/rosalindfranklininstitute/antigen-app>.

### Object storage

Using Amazon Web Services S3-compatible storage is recommended for production use. This is offered by many providers, including self-hosting using Minio. This option is configured in the AntigenApp backend environment variables.

### Backups

Backups are essential for production environments. This consists of backing up the PostgreSQL database and the object storage.

## References

Eyssen, L.E.- *et al.* (2024) ‘From Llama to Nanobody: A Streamlined Workflow for the Generation of Functionalised VHHs’, *BIO-PROTOCOL*, 14(1341). Available at: https://doi.org/10.21769/BioProtoc.4962.

Lubbock, A. *et al* (2025) AntigenApp: a laboratory data management system for nanobody generation and sequence analysis *Zenodo* October 20, 2025 *Version 0.1.* Available at https://doi.org/10.5281/zenodo.17397055
